# Foraging at Solid Urban Waste Disposal Sites as Risk Factor for Cephalosporin and Colistin Resistant *Escherichia coli* Carriage in White Storks (*Ciconia ciconia*)

**DOI:** 10.3389/fmicb.2020.01397

**Published:** 2020-07-28

**Authors:** Ursula Höfle, Juan Jose Gonzalez-Lopez, Maria Cruz Camacho, Marc Solà-Ginés, Albert Moreno-Mingorance, Jose Manuel Hernández, Javier De La Puente, Javier Pineda-Pampliega, José Ignacio Aguirre, Fernando Torres-Medina, Antoni Ramis, Natalia Majó, Julio Blas, Lourdes Migura-Garcia

**Affiliations:** ^1^SaBio (Health and Biotechnology) Working Group, Instituto de Investigación en Recursos Cinéticos IREC (CSIC-UCLM-JCCM), Ciudad Real, Spain; ^2^Servei de Microbiologia, Hospital Vall d’Hebron, Universitat Autònoma de Barcelona, Barcelona, Spain; ^3^IRTA, Centre de Recerca en Sanitat Animal (CReSA, IRTA-UAB), Universitat Autònoma de Barcelona (UAB), Bellaterra, Spain; ^4^Independent Researcher, Cañada de Calatrava, Spain; ^5^Bird Monitoring Unit, SEO/BirdLife, Madrid, Spain; ^6^Department of Biodiversity, Ecology and Evolution, Faculty of Biology, Complutense University of Madrid, Madrid, Spain; ^7^Departamento de Biología de la Conservación, Estación Biológica de Doñana, Consejo Superior de Investigaciones Científicas, Seville, Spain; ^8^Department of Biology, University of Saskatchewan, Saskatoon, SK, Canada; ^9^Departament de Sanitat i Anatomia Animals, Universitat Autònoma de Barcelona (UAB), Bellaterra, Spain

**Keywords:** *Escherichia coli*, virulence factors, white stork *Ciconia*, solid urban waste landfills, cephalosporin resistance

## Abstract

White stork (*Ciconia ciconia*) may act as a reservoir and vehicle of cephalosporin resistant (CR) *Escherichia coli*. Between 2011 and 2014, we sampled white storks from colonies exposed to different degrees of anthropic pressure across the major areas of natural distribution of white storks in Spain. Cloacal swab samples (*n* = 467) were obtained from individuals belonging to 12 different colonies from six different regions. Additionally, 70 samples were collected from recently deposited droppings at the base of nesting platforms. We phenotypically characterized *E. coli* isolates, confirmed presence of CR genes and classified plasmids. Risk factors for acquiring these genes were assessed. Overall, 8.8% (41 out of 467) storks carried CR *E. coli* in their cloaca and five (7.1%) were identified from recently deposited droppings; therefore, 46 isolates were further characterized. Of them, 20 contained *bla*_CTX–M–__1_, nine *bla*_CMY–__2_, six *bla*_CTX–M–__14_, four *bla*_SHV–__12_, three *bla*_CTX–M–__15_, two *bla*_CTX–M–__32_, one *bla*_CTX–M–__1_ together with *bla*_CMY–__2_, and one *bla*_CTX–M–__1_ together with *bla*_SHV–__12_. All were multidrug-resistant, and four harbored the plasmid-mediated colistin resistance *mcr-1* gene. CR genes were associated with the presence of IncI1, IncFIB, and IncN replicon families. *Xba*I-macrorestriction analysis revealed a great diversity among most of the *Xba*I-PFGE types, but indistinguishable types were also seen with isolates obtained from different locations. Clonal complex 10 was the most common among CR *E. coli* and two *bla*_CTX–M–__15_ positive isolates were identified as B2-ST131. Carriage of CR *E. coli* was significantly higher in colonies located close to solid urban waste disposal sites in which foraging on human waste was more likely and in one case to cattle grazing. The co-occurrence of *bla*_CMY–__2_ and *mcr*-1 on plasmids of *E. coli* isolated from wild birds as early as 2011 is of note, as the earliest previous report of *mcr*-1 in wild birds is from 2016. Our study shows that foraging at landfills and in association with cattle grazing are important risk factors for the acquisition of CR *E. coli* in white storks.

## Introduction

Over the past years, the presence of antimicrobial resistant bacteria in wildlife, particularly cephalosporin resistant (CR) *Escherichia coli* has become a problem of increasing concern in public health (Guenther et al., 2011). The genes encoding antimicrobial resistance are frequently located on plasmids, which may mediate the horizontal transfer of such genes to other bacteria (Carattoli, 2013; Dierikx et al., 2013). The overuse of antimicrobials in both human and veterinary medicine has been considered one of the main factors contributing to the dissemination of antimicrobial resistant bacteria, increasing their detection in humans, food-producing animals, and food (Jouini et al., 2007; Smet et al., 2010). Furthermore, antimicrobial residues from urban and livestock sources may persist over time in soil and aquatic environments (Kemper, 2008). As a consequence, the emergence of antimicrobial resistant bacteria in the environment may be facilitated (Martínez, 2009), representing a global threat of major concern for human, animal, and environmental health.

Some studies have demonstrated the presence of biologically active antibiotic residues in animal and human waste, such as sewage and manure (Kummerer, 2009; Cycoń et al., 2019; Menz et al., 2019). In the case of wild birds, most of the studies have associated the influence of human activities such as farming, presence of dumpsites, or even tourism with the detection of antibiotic resistant bacteria (Allen et al., 2010; Ahlstrom et al., 2019). In addition, several reports have suggested the importance of wildlife in the dissemination of CR *E. coli* (Pinto et al., 2010; Veldman et al., 2013; Báez et al., 2015). Since wild birds in their natural environment are not treated with antibiotics (Santos et al., 2013), they are potential sentinels of multidrug resistant bacteria discharged into the environment.

The role of wild birds as disseminators of antibiotic-resistant bacteria between distant ecosystems is difficult to estimate (Báez et al., 2015; Ahlstrom et al., 2019). White storks (*Ciconia ciconia*), like many other free-living migratory birds, can become long-distance vectors of CR *E. coli*. They feed nearby pastures and plowed fields, marshy wetlands, rice fields, and more recently on solid human waste disposal sites, and display long-distance movement patterns within and between continents (Flack et al., 2016; Bécares et al., 2019). Due to their mobility and contact with fecal contamination in pastures and surface waters, they may effectively acquire and spread disease and antimicrobial-resistant bacteria (Szczepanska et al., 2015) and become a potential source of CR *E. coli* for humans and farm animals (Tryjanowski et al., 2006; Keller et al., 2011). White storks may breed in open farmland with access to marshy wetlands but also, very often, they cohabit with humans, making use of manmade facilities such as roofs of buildings, telephone or electric power line poles, or other constructions for their nests. If such individuals acquire CR *E. coli* during feeding, they can act as potential reservoirs of resistant bacteria (Liakopoulos et al., 2016a, b).

There have been several studies reporting detection of CR *E. coli* in wild birds (Veldman et al., 2013; Zurfluh et al., 2013; Báez et al., 2015) and Alcalá et al. (2016) described for the first time the presence of CR *E. coli* in a white stork in Spain. However, to our knowledge this is the first study to analyze carriage of CR *E. coli* in white storks, in detail, in a large number of colonies subjected to different degrees of anthropic pressure with the aim of determining the epidemiology of CR *E. coli* in this species and their role as potential spreaders of CR *E. coli.* For this purpose, the isolates have been extensively characterized by antimicrobial susceptibility testing (AST), the identification of extended-spectrum β-lactamase (ESBL) and plasmid-mediated AmpC β-lactamase (pAmpC) genes, and the plasmids harboring these genes, phylotyping, genotypic relationships and the detection of virulence genes associated to avian pathogenic *E. coli* (APEC).

## Materials and Methods

### Ethics Statement

Sampling of white storks was in all cases associated to ringing or radio-tagging activities. None of the storks were specifically captured or handled for the purpose of this study. All ringing and radio-tagging activities were carried out under the pertinent permits from the local authorities (regional governments of Castilla – La Mancha, Madrid, Extremadura, Castilla y Leon, and Andalucía) and by registered specialized personnel. Handling and sampling of the storks was carried out following all applicable international, national, and/or institutional guidelines for the care and ethical use of animals, specifically directive 2010/63/EU and Spanish laws 9/2003 and 32/2007, and Royal decrees 178/2004 and 1201/2005.

### Field Sampling

**FIGURE 1:**
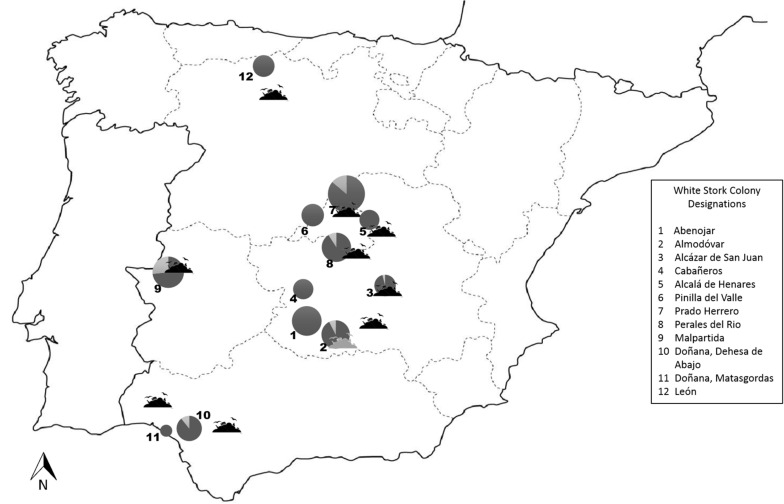
Distribution of studied colonies and the closest solid urban waste landfills in Spain. The size of the circles reflect sample size and the lighter shaded areas reflect CR *E. coli* prevalence. The shaded solid urban waste landfill silhouette shows a landfill that was sealed in 2005.

The study area covered most of the natural distribution of white storks in Spain. We collected cloacal swab samples (*n* = 467) from storks from 12 different colonies located in five different regions. Sampling occurred more intensively and in consecutive years in colonies situated in South-central Spain. Hence, six colonies in South-central Spain were sampled in 2013 and 2014. From two colonies, samples were obtained only in 2013 and four additional colonies were sampled only in 2014 (Figure 1 and Table 1). In addition, recently deposited droppings were recovered at the base of the nesting platforms in three of the six colonies in South-central Spain in 2011.

**TABLE 1:** Location, distance to closest human waste disposal site, presence of livestock, and type, and number of samples and presence of CR *E. coli* obtained from the different white stork colonies.

Colony designation	Province	Distance to human waste disposal site (km)	Live-stock	Year	Age	Number of samples	CR *E. coli*
Abenójar	Ciudad Real	60.95	Extensive sheep	2011	Droppings	23	0 (0%)
				2013	Nestling	28	0 (0%)
					Adult	2	0 (0%)
				2014	Nestling	29	0 (0%)

Almodóvar	Ciudad Real	50.58 (formerly 1 km)	Extensive sheep	2011	Droppings	10	0 (0%)
				2013	Nestling	30	2 (6.7%)
					Adult	2	0 (0%)
				2014	Nestling	30	3 (10%)

Alcázar de San Juan	Ciudad Real	0.77	Extensive sheep	2011	Droppings	37	5 (13.5%)
				2013	Nestling	8	0 (0%)
					Adult	3	0 (0%)
				2014	Nestling	13	1 (7.7%)

Cabañeros	Ciudad Real	64.91	No	2013	Nestling	15	0 (0%)
					Adult	2	0 (0%)
				2014	Nestling	19	0 (0%)

Alcalá de Henares	Madrid	2.609	No	2014	Nestling	14	0 (0%)

Pinilla del Valle	Madrid	30.417		2013	Nestling	31	0 (0%)
			Extensive cattle		Adult	2	0 (0%)
				2014	Nestling	29	0 (0%)

Prado Herrero	Madrid	11.425	Cattle (breeder)	2013	Nestling	30	6 (20%)
				2014	Nestling	57	8 (14%)

Perales del Rio	Madrid	6.343	No	2014	Nestling	20	2 (10%)

Malpartida	Cáceres	6.547	Extensive sheep	2013	Nestling	43	16 (37.2%)
					Adult	1	0 (0%)

Doñana, Dehesa de Abajo	Sevilla	33.203	Extensive cattle	2014	Nestling	18	1 (5.6%)
					Adult	7	2 (28.6%)

Doñana, Matasgordas	Huelva	40.370	Extensive cattle	2014	Adult	6	0 (0%)

León	León	38.279	Extensive cattle	2013	Nestling	28	0 (0%)
					Adult	1	0 (0%)

To take specific aspects of the feeding and spatial ecology of white storks into account, samples were obtained during the reproductive season (approximately February–July), when white storks are spatially bound to their colony/nest. During this period, foraging occurs mostly in a spatially defined area and can be linked fairly clearly to a specific type of habitat/food source, allowing for the comparison of the effect of feeding on natural and/or human origin (waste) resources (Alonso et al., 1991; Tortosa et al., 2002). In contrast, during the migration and wintering periods, the spatial distribution of foraging increases, as well as the intensity of use of human waste disposal sites as a predictable food source, making association of storks to a specific habitat more difficult (Ciach and Kruszyk, 2010; Bécares et al., 2019). The continuous predictable availability of food on landfills has a profound effect on the migratory patterns of several avian species including white storks (Plaza and Lambertucci, 2017). While juvenile white storks and some adult birds still migrate to Africa, the majority have shortened their migratory distance considerably. As a result, many Spanish white storks winter in Spain on, or close to, landfills and are joined by birds from central and northern Europe, that either are on stopover on their route toward Africa, or remain in Spain for the winter (Gilbert et al., 2016; Bécares et al., 2019).

Samples included, on one hand recently deposited droppings collected below nesting platforms in three colonies in South-central Spain (*n* = 70) in March 2011 (just prior to egg-laying), thus presumably belonging to the adults breeding on each particular platform. On the other hand, sampling comprised cloacal swab samples from adult and nestling white storks collected during the breeding period (May–July) in 2013 (*n* = 226) and 2014 (*n* = 241).

The largest part of the cloacal swab samples (one per bird *n* = 441) was collected from chicks during the ringing activities performed at their nests (nestlings). This allowed for the collection of a considerable number of samples without the need of stressful, costly and time-consuming capture. In addition, cloacal swab samples were obtained from 26 breeding adults captured for satellite/gps data logger transmitter fitting, in their nests (the presence of small chicks at their nest was certified), meaning that the samples were obtained in a period were foraging was also concentrated in a defined type of habitat. Tarsus length and body weight were recorded in all white storks in order to enable calculation of a scaled mass index (SMI), subsequently used as a proxy for body condition.

Recently deposited droppings were collected using sterile cotton tip swabs and small zip-lock bags. The samples were kept at 4°C until arrival at the laboratory and divided into a subsample for storage at −80°C and a sample for immediate processing. Cloacal swab samples were obtained using sterile cotton swabs in AMIES transport medium (Deltalab, Barcelona, Spain). They were kept at 4°C until arrival to the laboratory, where they were processed in less than 12 h after sampling. Samples were plated onto MacConkey agar (Oxoid, Basingstoke, United Kingdom) supplemented with 4 mg/L cefotaxime (Sigma-Aldrich Chemical, Madrid, Spain), and incubated overnight at 37°C. Lactose positive colonies morphologically compatible with *E. coli* were considered indicative of growth above breakpoint concentrations of cefotaxime based on the Clinical and Laboratory Standards Institute (CLSI) document, describing clinical resistance for cefotaxime ≥4 (CLSI, 2020). From these plates, three lactose-positive colonies were stored at −80°C in 30% glycerol BHI (Brain-Heart Infusion broth, Scharlau Microbiología, Barcelona, Spain). Subsequently, one representative colony was selected for further characterization in the present study and confirmed as *E. coli* by PCR (Heininger et al., 1999).

### Antimicrobial Susceptibility Testing

The combined disc method was used for the confirmatory tests for ESBL-producing *E. coli* (CLSI, 2020). Therefore, the following discs were used: cefotaxime, 30 μg, cefotaxime + clavulanic acid, 30 + 10 μg; ceftazidime, 30 μg; ceftazidime + clavulanic acid, 30 + 10 μg (Oxoid, Basingstoke, United Kingdom). For the identification of pAmpC-producing *E. coli*, the cefoxitin disc (30 μg) was used. Additionally, the cefepime disc (30 μg) was also used. Moreover, all isolates were subjected to susceptibility testing by broth microdilution (VetMIC GN-mo, National Veterinary Institute, Uppsala, Sweden) to the following antimicrobial agents: ampicillin (1–128 mg/L), cefotaxime (0.016–2 mg/L), ceftazidime (0.25–16 mg/L), nalidixic acid (1–128 mg/L), ciprofloxacin (0.008–1 mg/L), gentamicin (0.12–16 mg/L), streptomycin (2–256 mg/L), kanamycin (8–16 mg/L), chloramphenicol (2–64 mg/L), florfenicol (4–32 mg/L), trimethoprim (1–128 mg/L), sulfamethoxazole (8–1,024 mg/L), tetracycline (1–128 mg/L), and colistin (0.5–4 mg/L). The *E. coli* ATCC 25922 was used as a control strain. Isolates were considered to be wild-type or non-wild-type based on epidemiological cut-off (ECOFF) values defined by EUCAST^1^. ECOFF separates wild-type populations from isolates that have developed reduced susceptibility for the antimicrobial and may differ from clinical breakpoints used for clinical settings (EFSA, 2017).

### Antimicrobial Resistance Genes

All cefotaxime resistant (CR) isolates were tested by PCR methods for the presence of the *bla*_CTX–M_, *bla*_SHV_, *bla*_CMY–__1_, and *bla*_CMY–__2_ genes as previously described by Hasman et al. (2005). The isolates showing resistance to colistin were also tested for the presence of *mcr* genes (Rebelo et al., 2018). Sequencing of both strands of amplicons was performed.

### Phylogeny, Macrorestriction Analysis and Multi Locus Sequence Typing

The isolates were classified in phylogenetic groups (A, B1, B2, C, D, E, or F) by PCR, according to a method previously described by Clermont et al. (2000, 2013).

Genomic DNA macrorestriction analysis by pulsed-field gel electrophoresis (PFGE) was performed to determine the genomic relatedness among the isolates. The experiments were carried out as described in the PulseNet protocol (Ribot et al., 2006). *Salmonella enterica* serovar Branderup H9812 was used as a size marker. The results were analyzed by Fingerprinting II Informatix software (Applied Maths, Sint-Martens-Latem, Belgium). Isolates were considered to have a different PFGE-type when changes in at least one band were detected after digestion with *Xba*I. The analysis of the PFGE-types was carried out using the Dice similarity coefficient and the unweighted-pair group method with arithmetic averages (optimization of 1.25% and position tolerance of 1.25%).

Multi locus sequence typing (MLST) was performed according to the protocol and primers specified on the *E. coli* MLST website^2^ (Wirth et al., 2006). Sequences were analyzed using Vector NTI advance 11 (InforMax, Inc., Bethesda, MD, United States). The allelic profile and the sequence types (STs) were obtained through the electronic database at the *E. coli* MLST website.

Whole-genome sequencing (WGS) was performed with *E. coli* isolates which revealed a new allele sequence or new allele combination after the Sanger sequencing. WGS was performed on a MiSeq Instrument (Illumina, San Diego, CA, United States) using the Nextera DNA Flex Library Prep Kit (Illumina) and paired-end sequenced with a MiSeq Reagent kit v3 (Illumina) with an average insertion size of 300 bp. The obtained sequences were submitted to Enterobase^3^ to obtain new allele/STs designation.

### Detection of Virulence-Associated Genes

**FIGURE 2:**
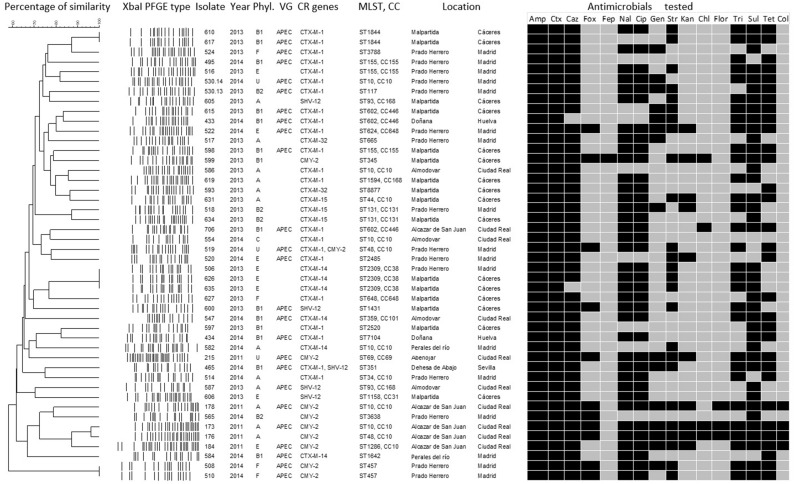
Phenotypic and genotypic characteristics of the cephalosporin-resistant *E. coli* isolates: dendrogram showing the genotypic relationships of the different *Xba*I-types of the isolates. Further characteristics are also shown: year of isolation phylogroup (Phyl. U, unidentified phylogroup), virulence-associated genes (VG) and cephalosporin and colistin resistance genes (CR), sequence type (ST); clonal complex (CC) and sampling location. The antimicrobial agents tested are also displayed and the black boxes below their abbreviated designations are indications for a non-wild-type phenotype. Amp, ampicillin (WT ≤ 8 mg/L); Ctx, cefotaxime (WT ≤ 0.25 mg/L); Caz, ceftazidime (WT ≤ 0.5 mg/L); Fox, cefoxitin (WT ≤ 8 mg/L); Fep, cefepime (WT ≤ 0.125 mg/L); Nal, nalidixic acid (WT ≤ 16 mg/L); Cip, ciprofloxacin (WT ≤ 0.064 mg/L); Gen, gentamicin (WT ≤ 2 mg/L); Str, streptomycin (WT ≤ 16 mg/L); Kan, kanamycin (WT ≤ 8 mg/L); Chl, chloramphenicol (WT ≤ 16 mg/L); Ff, florfenicol (WT ≤ 16 mg/L); Tm, trimethoprim (WT ≤ 2 mg/L); Sul, sulfamethoxazole (WT ≤ 64 mg/L); Tet, tetracycline (WT ≤ 8 mg/L); Col, colistin (WT ≤ 2 mg/L).

All 46 isolates were tested by multiplex PCR for the genes described previously by Johnson et al. (2008) as the minimal predictors of APEC virulence; *iroN*, *ompT*, *hlyF*, *iutA*, and *iss* (Figure 2).

### Mating Experiments and Plasmid Characterization

Filter mating experiments were carried out to assess the transferability of ESBL-encoding plasmids by conjugation. For the analysis, one representative isolate from each PFGE-type was selected. Mating assays were performed as previously described (Bielak et al., 2011), using the isolates as donors and rifampicin-resistant *E. coli* HB101 as a recipient. Transconjugants were selected on LB agar plates supplemented with rifampicin (150 mg/L) and ceftriaxone (1 mg/L). The identity of the transconjugants was confirmed by PFGE and the presence of the corresponding CR gene was confirmed by PCR.

Plasmid DNA was purified from the subset of 42 isolates (one isolate as representative of PFGE-type) using a Qiagen Plasmid Midi kit (Qiagen, Hilden, Germany) according to the manufacturer’s recommendations. Plasmids were introduced to electrocompetent plasmid free *E. coli* BL21 cells by electroporation. Transformants were selected in LB agar plates containing ceftriaxone (1 mg/L), and PCR assays were performed to confirm the presence of ESBL and/or pAmpC genes. The presence of a unique plasmid in the transformants and their corresponding sizes were analyzed using S1 nuclease digestion followed by PFGE (S1-PFGE) (Barton et al., 1995). Additionally, plasmids were classified by PCR-based replicon typing (Carattoli et al., 2005).

### Statistical Analysis

As a proxy to the degree of exposure of storks to human waste foraged food, we used the distance of each colony to the nearest human waste disposal site. This is based on the reduced home range of storks during chick raising and incubation and the assumption that storks are less likely to undertake long foraging flights during this period as compared to other stages. This hypothesis is supported by satellite transmitter data from radio-tagged adult storks from colonies in South-central Spain that show distinct foraging either in natural habitat or at human waste disposal sites (Bécares et al., 2019). In addition, the distance between a stork’s nest and solid human waste landfills has been shown to correlate with the frequency and intensity with which the storks forage on waste (Djerdali et al., 2016; Gilbert et al., 2016).

For this, the coordinates for each colony/nest sampled, in parallel to the coordinates of all known human waste disposal sites, were projected onto a scaled flat reference system in the geographic information system QGIS (QGIS desktop 2.8.1), and the direct flight distance to the nearest human waste disposal site (known to be used by storks) was calculated.

We calculated the body condition of white stork nestlings and adults sampled in the field according to the SMI proposed by Peig and Green (2009). This index can be computed as: *M* = *M*_*i*_ (*L*_0_/*L*_*i*_)^bsma^, where *M*_*i*_ and *L*_*i*_ are the body mass and the structural size measurement (tarsus length) of each individual respectively; bsma is the scaling exponent estimated by the standardized major axis (SMA); regression of *M* on *L*, *L*_0__^´´_ is the arithmetic mean value for the study population; *M* is the predicted body mass for individual *i* when the structural size body measure is standardized to *L*_0_. The scaling exponent “bsma” has been calculated indirectly by dividing the slope from an ordinary least squares (OLS) regression (bols) by the Pearson’s correlation coefficient *r*. Normal distribution of the continuous variable body condition index *M* was confirmed using the Kolmogórov–Smirnov test prior to further analysis. We compared body condition between ESBL and APEC “infected” and “uninfected” nestlings using Student’s *t*-test and the effect of distance of colonies to solid human waste landfills on SMI as a proxy of body condition using a one-way ANOVA. We employed a generalized linear mixed model (GLMM) with a binary response and a logit regression, and the nest of origin within the colony as random factor, to determine the effect of sampling year, sex, number of siblings in the nest, presence of livestock (absence, extensive small ruminant, and cattle grazing) and the distance to human waste disposal sites on the prevalence of CR *E. coli*, in white stork nestlings. All analyses were carried out using SPSS statistical software, version 24.0 (IBM^®^, SPSS Inc., Chicago, IL, United States) and significance was set at *p* ≤ 0.05.

## Results

### Prevalence of CR *E. coli*, AST and Resistance Genes

Overall CR *E. coli* prevalence in cloacal swab samples was 8.8% (41 out of 467). Five (7.1%) of the isolates from recently deposited droppings (*n* = 70) were identified as CR *E. coli*, but, in contrast to cloacal swab samples, we cannot unequivocally associate the droppings to specific individuals. For this reason, statistical analysis concerning CR *E. coli* was only carried out on results from cloacal swab samples.

Extended-spectrum β-lactamase and/or presumptive pAmpC phenotypes were confirmed for all 46 CR isolates. These 46 isolates were also multidrug-resistant (resistant, according to ECOFFs, to at least three antimicrobial families). Furthermore, 54% (25/46) exhibited a non-wild-type phenotype to more than seven antimicrobial classes. MIC values determined that all isolates exhibited a non-wild-type phenotype to cephalosporins (100% resistance to cefotaxime and 96% to ceftazidime), with 20 isolates yielding amplicons for *bla*_CTX–M–__1_, nine *bla*_CMY–__2_, six *bla*_CTX–M–__14_, four *bla*_SHV–__12_, three *bla*_CTX–M–__15_, two *bla*_CTX–M–__32_, one *bla*_CTX–M–__1_ together with *bla*_CMY–__2_, and one *bla*_CTX–M–__1_ together with *bla*_SHV–__12_. Additionally, 100% of the isolates were non-wild-type to ampicillin, 85% to sulfamethoxazole, 80% to ciprofloxacin and nalidixic acid, 67% to tetracycline, 65% to trimethoprim, 54% to streptomycin, 33% to gentamicin, 22% to kanamycin, 9% to chloramphenicol, and 7% to florfenicol (Figure 2).

In addition, four isolates from recently deposited droppings collected at nests located at the colony closest to a landfill (Alcázar de San Juan, Table 1) were phenotypically resistant to colistin (MIC ≥ 4) and were confirmed to carry the *mcr-1* gene (id. 173, 176, 178, and 184).

### Phylogeny, PFGE, and MLST

The phylotyping analysis identified six different phylogenetic groups among the 46 isolates (Figure 2). Of them, 14 belonged to phylogroup B1 (30%), 12 to group A (26%), eight to group E (17%), four to group B2 (9%), four to group F (9%), and one to group C (2%). No phylogroup could be determined for three of the isolates (7%).

Among the 46 CR *E. coli* isolates, 42 different macro-restriction profiles were detected. However, during 2013, a cluster of isolates sharing a similar PFGE profile (100% similarity) and identical MLST type (ST2309) was identified. These isolates (506, 626, and 635) were obtained from two different colonies located within a radius of about 250 km, in Madrid and Cáceres.

The MLST analysis also revealed a high degree of diversity. Overall, 30 different STs were found among the 46 isolates (mean of number of isolates per ST, 1.53; range, 1–6) (Figure 2). ST10 was the most prevalent type (6 isolates; 13%), followed by ST155, ST602, and ST2309 (3 isolates; 6.5%, each) and by ST48, ST93, ST131, ST457, and ST1844 (2 isolates; 4.3%, each). ST8877 was firstly identified in this study. Grouping STs by clonal complexes (CC) showed that CC10 was the most prevalent including 22% of the isolates, followed by CC38, CC155, CC168, and CC446 in the 3% of the population respectively (Figure 2).

### Detection of Virulence Genes Associated to APEC

Out of 46 isolates, 70, 70, 70, 57, and 52% yielded amplicons for *ompT*, *hlyF*, *iutA*, *iss*, and *iroN*, respectively. Accordingly, 25 isolates (54%) were considered APEC, since they had four to five of the genes mentioned above (Figure 2).

### Conjugation and Transformation Experiments

Among the 46 CR isolates, a subset of 42 representing each *Xba*I-macrorestriction profile were selected for conjugation and transformation experiments. Of them, 15 were able to transfer the CR genes by conjugation. Additionally, 33 transferred the CR genes to the BL21 electrocompetent strain. S1-nuclease PFGE confirmed the presence of a unique plasmid in all selected isolates except in two of them (518 and 598), which contained more than one plasmid. Sizes of plasmids varied between 40 and 150 kb approximately (Table 2). PCR-based replicon typing showed that IncI1 was the most common replicon and was associated to *bla*_CTX–M–__1_, *bla*_CTX–M–__14_, *bla*_CMY–__2_, and *bla*_SHV–__12_ genes. IncI1 was present in 25 of the isolates, followed by IncFIB and IncN. The replicons IncFIA, IncFIC, IncA/C, IncK, and IncL/M were also detected (Table 2). Four isolates presented between two and five replicons on the same plasmid (517, 631, 176, and 184), and none of the tested replicons were detected in seven of the transformants or transconjugants (434, 514, 554, 508, 173, 626, and 634).

**TABLE 2:** Characteristics of plasmids harboring ESBL and/or pAmpC genes from 42 cephalosporin-resistant *E. coli* isolates.

Gene and isolate	Conjugation result	Transformation result	Inc type(s)								Plasmid size (kb)
			I1	FIB	N	K	FIA	FIC	A/C	L/M	
*bla*_CTX–M–__1_											
516	**TC1b**		+								100
524	**TC3a**		+								90
530.13		**TF4a**	+								60
586	**TC5a**	TF5a	+								100
598	–	–									
597	TC7a	**TF7a**	+								100
610		**TF9a**	+								100
615		**TF10b**	+								90
619		**TF11a**	+								100
627		**TF12a**	+								100
706		**TF14a**	+								100
433		**TF15a**	+								110
434	**TC16b**	TF16a									110
495	TC17a	**TF17a**		+							50
514		**TF18a**									60
520		**TF19a**	+								100
522		**TF38a**	+								110
530.14		**TF20a**			+						40
554	TC21a	**TF21a**									50
*bla*_CTX–M–__32_											
517		**TF2a**		+				+	+		130
593		**TF6a**	+								100
*bla*_CTX–M–__14_											
626		**TF30a**									110
547		**TF31a**	+								110
582	TC32a	**TF32a**				+					80
584	TC33a	**TF33a**	+								90
*bla*_CTX–M–__15_											
631		**TF13a**		+			+				150
634	**TC39b**										60
518	–	–									
*bla*_CMY–__2_											
599		**TF22a**							+		120
508		**TF23a**									100
565	**TC24a**	TF24a	+								80
173		**TF25a**									90
176		**TF26a**	+	+	+			+		+	90
178		**TF27a**	+								90
184	**TC28b**		+		+	+					40
215		**TF29b**			+						40
*bla*_SHV–__12_											
587	**TC34a**		+								90
600	**TC35c**		+								90
605		**TF36a**	+								100
606		**TF8b**	+								100
*bla_CTX–M–__1_ + *bla*_CMY–__2_											
519		**TF37a**	+								100
*bla_CTX–M–1_ + *bla*_SHV–12_											
465	**TC40a**			+							110

*Transconjugants (TC) and transformants (TF) used for PCR-based replicon typing method are in boldface. Two isolates (518 and 598) presented more than one plasmid in the TC and/or TF isolates), therefore they are not included in the table. ^∗^Only *bla*_CTX–M–__1_ was transferred to the transformant and transconjugant isolate, respectively.*

### Risk Factors for the Acquisition of CR *E. coli* by White Storks

Cephalosporin resistant *E. coli* prevalence did not vary significantly between nestling (8.8%, 39 out of 441) and adult storks (7.7%, 2 out of 26). Sampling year [*F*(1,359) = 2.538, *p* = 0.112], Sex [*F*(1,359) = 0.517, *p* = 0.473], number of siblings in the nest [*F*(1,359) = 1.645, *p* = 0.179], and livestock grazing close to the colony [*F*(2,359) = 1.78, *p* = 0.17] did not affect CR *E. coli* carriage in white stork nestlings. However, distance of the colony to human waste disposal sites had a significant effect on the prevalence of CR *E. coli* [GLMM *F*(1,359) = 8.641, *p* = 0.004], with nestlings from colonies close to landfills being significantly more likely to carry CR *E. coli* (Figure 1 and Table 3). Carriage of CR *E. coli* [Student’s *t*-test *F*(1,359) = 2.946, *p* = 0.258] or APEC [Student’s *t*-test *F*(1,359) = 1.921, *p* = 0.164] had no significant effect on the body condition of white stork nestlings. However, white stork nestlings from colonies close to human waste disposal sites were in significantly better body condition than nestlings from colonies far from human waste disposal sites [ANOVA *F*(7,341) = 8.469, *p* < 0.001].

**TABLE 3:** Results of generalized mixed linear model (GLMM) for cephalosporin resistant (CR) *E. coli* carriage in white stork nestlings in Spain.

Model	Predictor	Test model effects	Description	*F*	Standard Error	*t*	*p*
CR *E. coli*	Livestock presence	0.304	None	0.468	1.121	0.418	0.677
			Ext sheep	–1.08	0.758	–1.424	0.155
			Ext cattle	0	–	–	–
	Sex	0.170	F	0.319	0.444	0.719	0.473
			M	0	–	–	–
	Sampling year	0.112	2013	–0.921	0.578	–1.593	0.112
			2014	0	–	**–**	**–**
	Distance landfill (m)	**0.004**	Continuous	0.000	0.000	2.909	**0.004**
	Number of siblings in nest	0.179	Single chick	1.729	0.921	1.877	0.061
			Two siblings	1.54	0.775	1.988	**0.048**
			Three siblings	0.816	0.661	1.234	0.218
			Four siblings	0	–	–	–

*Statistically significant results are highlighted in bold.*

## Discussion

White storks are free-living colonial birds that frequently use humanized habitats. In the recent past, they have adapted to foraging at solid urban waste disposal sites and introduced American crayfish (*Procambarus clarkii*) in rice fields and small water bodies as predictable food sources during winter. This has led to a significant modification of migratory behavior of a large part of the adult stork population of Western Europe as well as, in the case of solid urban waste disposal sites, to the exposure to numerous contaminants and pathogens (Flack et al., 2016; Gilbert et al., 2016).

As storks cohabit in the proximities of humans and food producing animals, they are able to acquire bacteria from these and transport them to other locations in their annual or regular movements (Tryjanowski et al., 2006). The present study takes advantage of the fact that even though during winter most storks forage on human waste; during breeding the nesting site and colony determine the foraging ecology and allow for the characterization of risk factors for the exposure of white storks to CR *E. coli* (Alonso et al., 1991; Tortosa et al., 2002; Bécares et al., 2019). Our results suggest that the stork nestlings more likely fed human waste by their parents (using distance to rubbish dump as a proxy) were more likely to carry CR *E. coli*. CR *E. coli* carriage did not affect body condition in “infected” nestlings. Hence, the present study has demonstrated the presence of CR *E. coli* in white storks feeding on human waste and their capacity to disseminate and transport the resistant bacteria. In particular, similar clones with the same resistance genes have been detected in nestlings from colonies in Madrid and Caceres, located within a 250 km distance. These results suggest a common source of contamination, together with the capacity of the birds to spread the same clones during their movements. In this context, a colony that is now at a more than 50 km flight distance from the closest human waste disposal site, was formerly situated less than 1 km from a different disposal site that was sealed in 2005, but satellite transmitter data from two adults evidenced occasional visits to this landfill even during the breeding season (Table 1 and Figure 1). According to our results, extensive livestock farming in natural habitats (present in the proximity of at least five of six natural habitat colonies) appears to be less of a risk factor for the acquisition of CR *E. coli* by white storks. However, nestlings from one colony located on a farm that raises cattle and where storks and cattle share the pastures, also situated at a flight distance of 11 km to the nearest landfill (Prado Herrero, Table 1), most commonly carried CR *E. coli* with the *bla*_CTX–M–__1_ gene, which is commonly described in cattle in Europe (Schmid et al., 2013). Storks frequently collect cattle dung and place it in the nest just prior to, and a few weeks after hatching, presumably to help chicks maintain body temperature (Tortosa and Villafuerte, 1999). This behavior as well as foraging of the adults on contaminated pastures (especially on dung beetles) is a likely route of exposure of the nestlings to CR *E. coli* excreted by cattle.

Some studies suggest that wildlife also play an important role in the global dissemination of antimicrobial resistance genes, transferring mobile genetic elements between bacterial communities (Allen et al., 2010). In our study, the presence of multidrug-resistant *E. coli* demonstrated the potential capacity of white stork to disseminate and transport ESBL and/or AmpC encoding genes located in conjugative plasmids. These isolates were all resistant to third generation cephalosporins and most of them were also resistant to fluoroquinolones. These two families of antimicrobials are nominated of critical importance in human health since they are widely used for the treatment of human infections by Gram-negative bacteria (European Centre for Disease Prevention and Control [ECDC], 2018). In addition, four of the isolates, obtained in 2011 from recently deposited droppings, co-harbored *bla*_CMY–__2_ and *mcr-1*, although on different plasmids. They were also resistant to multiple antibiotics including third-generation cephalosporins, fluoroquinolones, colistin, and aminoglycosides, among others, making these isolates resistant to almost all last-line antimicrobials available. Interestingly, all *mcr-1* carrying isolates presented different PFGE profiles and belonged to the CC10, two were ST10, one was ST48 and one was ST1286. This high risk CC has previously been reported as the most commonly associated with *mcr-1* globally, not only in humans but also in animals and in environmental samples (Caltagirone et al., 2017; Matamoros et al., 2017; García-Meniño et al., 2018; Lalaoui et al., 2019). All STs found within this study, except for ST8877, have previously been identified in third-generation CR isolates of animal origin (Dierikx et al., 2013; Solà-Ginés et al., 2015; Alcalá et al., 2016; Batalha de Jesus et al., 2019). Of note, CR *E. coli* CC10 has been reported as one of the most prevalent lineages on farms, food, and waste water plants, and the second most abundant in humans in Spain (Ojer-Usoz et al., 2017). Additionally, ST131, ST69, and ST10 are the most prevalent ExPEC lineages found in humans (Manges et al., 2019), and all of them have also been identified in this study. This fact also highlights the importance of wildlife as potential reservoirs, not only of antimicrobial resistance genes, but also of human pathogenic bacteria belonging to high-risk clones.

Several studies have suggested a relationship between the different *E. coli* phylogroups and the virulence of the isolates (Picard et al., 1999; Clermont et al., 2011). Accordingly, non-virulent isolates mainly belong to A, B1, and C phylogenetic groups, whereas the most virulent phylogroups are associated to B2 followed by D (Russo and Johnson, 2000). In this study, most of the isolates belonged to phylogroups A and B1, and only four isolates were B2. On the contrary, analyzing phylogroups and virulence genes in this strain collection, we have observed that almost all B1 isolates had the APEC virulence-associated genes, whereas only one B2 strain had the APEC genes. This may be due to the presence of virulence factors associated to mobile genetic elements such as in the case of the previously described ColV plasmid (Johnson and Nolan, 2009), which contained several virulence genes within the same plasmid (*hlyF*, *ompT*, *iss*, and *cvaC*). Therefore, the acquisition of such type of plasmid may confer B1 isolates with more virulence factors than expected. Carriage of *E. coli* isolates harboring APEC-associated genes in the cloaca of white stork nestlings was apparently not related to solid urban waste disposal site origin of food or any of the other factors; neither did it affect body condition.

The present study showed a high variability of CR genes among the isolates, *bla*_CTX–M–__1_ being the most prevalent (48%), followed by *bla*_CMY–__2_ and *bla*_CTX–M–__14_ (20 and 13%, respectively). These *bla*_CTX–M_ types are the most frequent variants in ESBL producers in animals and food of animal origin, while *bla*_CTX–M–__15_ has been mostly described in human medicine (Valverde et al., 2015) and occasionally on farms (Cameron-Veas et al., 2015). This study has demonstrated the presence of the *bla*_CTX–M–__15_ gene in three isolates of white stork harbored in plasmids of different sizes and in one occasion contained in an IncFIB replicon, the most common incompatibility group for *bla*_CTX–M–__15_ isolated from humans in Spain (Carattoli, 2009). Additionally, the remaining two *bla*_CTX–M–__15_ isolates were B2-ST131. They were obtained from two different colonies, one of which is closely associated to a village with part of the nests on urban structures, and confirmed consumption of human residues. The other colony is in a natural environment with no confirmed consumption of human residues during the breeding season, nevertheless this colony is situated at 30.4 km flight distance from Madrid, and thus exposure to human waste cannot be excluded completely. In fact satellite transmitter data from an adult stork from this colony shows extensive use of solid urban waste disposal sites outside the breeding period (Bécares et al., 2019).

Significant differences were found between location of the colonies and presence of CR genes. For instance, in the colony located in Caceres, the most prevalent gene was *bla*_CTX–M–__1_ associated to 90 and 100 kb plasmids, whereas in the colony located in Alcázar de San Juan (Ciudad Real) the most prevalent was *bla*_CMY–__2_ harbored in two types of plasmids (40 and 90 kb). The former was also the colony in which nestlings carried the greatest variety of CR genes (5) as compared to the other colonies.

The most common replicons found in the study were IncI1 (60%), followed by IncFIB (12%), and IncN (10%). To date, these three replicons are the most commonly found in plasmids carrying CR genes in *E. coli* isolated from poultry (Wang et al., 2013; Solà-Ginés et al., 2015). Almost all of the CR genes containing IncI1 replicons were located in plasmids of the same sizes, between 90 and 110 kb; specifically, 100 kb in the case of *bla*_CTX–M–__1_. However, in the case of two isolates, isolation of the plasmid carrying the CR gene could not be achieved. During transformation and conjugation experiments, co-transfer of plasmids of different molecular weights was observed. The entry of two or more plasmids into a single transformation procedure is not uncommon; Weston et al. (1979) demonstrated that 20% of the transformants could be double transformants (Weston et al., 1979). This type of transformation can be frequent if it is performed with high amounts of plasmid DNA (Goldsmith et al., 2007).

## Conclusion

In conclusion, results from this study confirm that exposure of white storks to human waste constitutes a risk factor for the acquisition of CR genes as the carriage of CR *E. coli* was significantly higher in colonies located close to solid urban waste disposal sites. As all individuals included in this study are free-living birds, they have not been treated with antimicrobials. Since they are not subjected to any direct antibiotic pressure and they cohabit in the proximities of humans, the presence of these resistance traits is a reflection of the anthropic pressure of human activities and particularly exposure to human waste and farming practices. In fact, outside the breeding season adult and juvenile storks have been observed to use human waste disposal sites indistinctive of the habitat of the colony of origin (Bécares et al., 2019). The distinctive pattern of prevalence of CR genes in white stork nestlings in association to foraging behavior of their parents during the breeding season, in addition to recent findings that show that a large proportion of storks are exposed to human waste from solid urban waste landfills during wintering, suggests that persistence of CR genes in bacteria of the digestive tract of white storks is relatively short. This makes white storks excellent sentinels for local antibiotic pressure in their foraging habitat. Nevertheless, due to their great mobility and migratory habits, the white stork can also act as carriers of CR *E. coli*, contributing to the global dissemination of antimicrobial resistance genes and plasmids.

## Data Availability Statement

The raw data supporting the conclusions of this article will be made available by the authors, without undue reservation, to any qualified researcher.

## Ethics Statement

Ethical review and approval was not required for the animal study because handling of the storks included in the study had been approved for the specific ongoing studies in each colony, and our study was based on fecal droppings or cloacal swabs only.

## Author Contributions

MC, AR, NM, and UH designed the study. UH, MC, JM, JD, JP-P, JA, FT-M, and JB sampled and collected relevant biological data from the stork colonies and isolated the isolates for further characterization. MS-G and LM-G carried out the identification, phenotypic, and molecular characterization of isolates and plasmids. JJ and AM-M performed MLST and WGS. UH and MC performed the statistical analysis. UH, MS-G, and LM-G drafted the manuscript. All authors revised the draft manuscript and contributed to writing the final version of the manuscript.

## Conflict of Interest

The authors declare that the research was conducted in the absence of any commercial or financial relationships that could be construed as a potential conflict of interest.

## Footnotes

1 http://www.eucast.org/

2 http://mlst.warwick.ac.uk/mlst/dbs/Ecoli

3 http://enterobase.warwick.ac.uk/
